# Can Inoculation With the Bacterial Biostimulant *Enterobacter* sp. Strain 15S Be an Approach for the Smarter P Fertilization of Maize and Cucumber Plants?

**DOI:** 10.3389/fpls.2021.719873

**Published:** 2021-08-24

**Authors:** Mónica Yorlady Alzate Zuluaga, André Luiz Martinez de Oliveira, Fabio Valentinuzzi, Raphael Tiziani, Youry Pii, Tanja Mimmo, Stefano Cesco

**Affiliations:** ^1^Faculty of Science and Technology, Free University of Bolzano, Bolzano, Italy; ^2^Department of Biochemistry and Biotechnology, State University of Londrina, Londrina, Brazil

**Keywords:** *Zea mays*, *Cucumis sativus*, plant growth promoting bacteria, root exudation, P-solubilisation, phosphorus transporter, gene expression

## Abstract

Phosphorus (P) is an essential nutrient for plants. The use of plant growth-promoting bacteria (PGPB) may also improve plant development and enhance nutrient availability, thus providing a promising alternative or supplement to chemical fertilizers. This study aimed to evaluate the effectiveness of *Enterobacter* sp. strain 15S in improving the growth and P acquisition of maize (monocot) and cucumber (dicot) plants under P-deficient hydroponic conditions, either by itself or by solubilizing an external source of inorganic phosphate (Pi) [Ca_3_(PO_4_)_2_]. The inoculation with *Enterobacter* 15S elicited different effects on the root architecture and biomass of cucumber and maize depending on the P supply. Under sufficient P, the bacterium induced a positive effect on the whole root system architecture of both plants. However, under P deficiency, the bacterium in combination with Ca_3_(PO_4_)_2_ induced a more remarkable effect on cucumber, while the bacterium alone was better in improving the root system of maize compared to non-inoculated plants. In P-deficient plants, bacterial inoculation also led to a chlorophyll content [soil-plant analysis development (SPAD) index] like that in P-sufficient plants (*p* < 0.05). Regarding P nutrition, the ionomic analysis indicated that inoculation with *Enterobacter* 15S increased the allocation of P in roots (+31%) and shoots (+53%) of cucumber plants grown in a P-free nutrient solution (NS) supplemented with the external insoluble phosphate, whereas maize plants inoculated with the bacterium alone showed a higher content of P only in roots (36%) but not in shoots. Furthermore, in P-deficient cucumber plants, all Pi transporter genes (*CsPT1.3, CsPT1.4, CsPT1.9*, and *Cucsa383630.1*) were upregulated by the bacterium inoculation, whereas, in P-deficient maize plants, the expression of *ZmPT1* and *ZmPT5* was downregulated by the bacterial inoculation. Taken together, these results suggest that, in its interaction with P-deficient cucumber plants, *Enterobacter* strain 15S might have solubilized the Ca_3_(PO_4_)_2_ to help the plants overcome P deficiency, while the association of maize plants with the bacterium might have triggered a different mechanism affecting plant metabolism. Thus, the mechanisms by which *Enterobacter* 15S improves plant growth and P nutrition are dependent on crop and nutrient status.

## Introduction

Crops cultivated around the world, belonging to both monocot and dicot clades, can be very diverse; nevertheless, their growth and development are strongly influenced by the state and the availability of nutrients in the soil. Consequently, the yield levels and the overall performance of a crop can considerably vary both spatially and temporally, depending on both soil characteristics and agricultural practices. In this context, phosphorus (P) is known to play a crucial role in plant nutrition; thus helping to achieve optimal growth and productivity. Plants take up P as inorganic phosphate (Pi); however, due to its low solubility and mobility, P is sparingly available in the soil solution, as its concentration is often rather limited (<10 μM) (Wang et al., 2017). To cope with this issue, plants have evolved morphological, biochemical, and metabolic strategies, which include: (i) the enhancement of root density and length, (ii) the release of organic acids and phosphatases, (iii) an increase in the expression of high-affinity Pi transporters, and (iv) the establishment of a symbiotic association with mycorrhizal fungi (López-Arredondo et al., 2014).

In addition to the genetic improvement of crops achieved through breeding programs, the exogenous root-inoculation with plant growth-promoting bacteria (PGPB) could prove itself to be an interesting and promising agricultural approach to the amelioration of the mineral nutrition of plants (Ramaekers et al., 2010). Indeed, beneficial bacteria colonize the root surface in the rhizosphere, where they can exert positive effects on a wide range of plant species *via* direct and indirect mechanisms (Dakora et al., 2015; Backer et al., 2018). The direct effects of PGPB inoculation can be related to either activity aimed at ameliorating the mineral nutrition of plants [e.g., nitrogen (N) fixation, P solubilization, the release of siderophores, and the enhancement of mineral nutrients uptake] (Pii et al., 2015b) or the release of hormone-like compounds that can modulate the growth of plants (Glick, 2012; Goswami et al., 2016). On the other hand, indirect effects are related to the ability of PGPB to protect plants from both abiotic (e.g., drought and salinity) and biotic (e.g., pathogens) stresses (Glick, 2012; Goswami et al., 2016).

Focusing on the biogeochemical cycle of P in soil, it has been well-demonstrated that PGPB can enhance the available fraction of this nutrient *via* an augmented secretion in the rhizosphere of organic acids and phosphatases (Billah et al., 2019). Although examples of improved plant acquisition of P have been well-described as a consequence of root inoculation with different PGPB strains, the mechanisms underlying the phenomenon remain unknown. The understanding of these interactions can be further complicated by the co-occurrence of processes underpinning P acquisition by plants; for instance, the increased nutrient bioavailability at the root-soil interface on the one hand, and the enhanced capacity of plants to transport Pi across the plasma membrane on the other hand (Pii et al., 2015b). Moreover, the ability of the roots to reacquire a wide range of small organic molecules belonging to root exudates, recently demonstrated in P-deficient tomato plants, further complicates the biochemical P cycle in the rhizosphere (Tiziani et al., 2020). Collectively, deeper comprehension of all these processes through the application of multidisciplinary approaches appears to be crucial to better exploit the properties of the PGPB strains at the field scale for the more efficient use of the endogenous P sources in soil within the context of more sustainable agriculture.

With respect to Pi-uptake mechanisms in roots, there are diverse experiences in literature aimed at the identification of the genes encoding the specific transporters and the characterization of the regulation of the entire Pi-uptake process in the roots (López-Arredondo et al., 2014). Considering the first aspect, among the genes identified, the *PHT1* gene family has the largest number of members expressed in roots, is well-recognized as mediators of Pi uptake from the external medium, and is predominantly overexpressed under P starvation (Wang et al., 2018). Considering crops and in particular monocots, 13 *PHT1* members have been identified in rice (*Oryza sativa*) (Paszkowski et al., 2002) and maize (*Zea mays*) (Liu et al., 2016), 16 in wheat (*Triticum aestivum*) (Grün et al., 2018), and nine in sorghum (*Sorghum bicolor*) (Tavares de Oliveira Melo, 2016). On the other hand, in dicots, 15 genes have been identified in the soybean (*Glycine max*) genome (Fan et al., 2013), eight in tomato (*Solanum lycopersicum*) (Chen et al., 2014) and potato (*Solanum tuberosum*) (Liu et al., 2017), and five in tobacco (*Nicotiana tabacum*) and pepper (*Capsicum frutescens*) (Chen et al., 2007). In this respect, it is interesting to note that studies aimed at characterizing the expression levels of these genes in inoculated plants (and, thence, the functionality of the Pi transporters) have been mainly conducted in plants colonized with arbuscular mycorrhizal fungi (AMF), while only a few of them considered inoculation with PGPB (Duan et al., 2015; Liu et al., 2018; Zhang et al., 2019). Therefore, the regulatory effects of PGPB on these transporters are still quite unknown. Additionally, considering that the effects of PGPB on plants significantly depend on specific interactions between plant genotypes and bacterial strains (Pii et al., 2015b; Crecchio et al., 2018), deeper knowledge about the PGPB strains (already isolated and belonging to the microbial collection banks in several research centers but not yet thoroughly investigated and characterized) appears to be fundamental.

Considering the possibility of improving P nutrition in crops using PGPB, it is worth mentioning that, in the context of previous research, the bacterium *Enterobacter* sp. strain 15S has been isolated and characterized for its particular ability to solubilize P *in vitro*, thus making it a promising tool for sustainable fertilization strategies (Zuluaga et al., 2020). Nevertheless, considering the abovementioned specificity in the PGPB-host interplay, it is mandatory to characterize the interaction of *Enterobacter* sp. strain 15S with model crops belonging to both monocots and dicots to unravel whether this bacterial strain might find a useful application at the field scale.

Based on these observations, the present research aimed to investigate the effect of *Enterobacter* sp. strain 15S inoculation on the development and P nutrition of cucumber and maize plants, which were chosen as representatives of the clades dicots and monocots, respectively. Considering the strong ability of P solubilization featured by *Enterobacter* sp. strain 15S, the bacterium effects on root P uptake of plants fed with an inorganic Pi source [Ca_3_(PO_4_)_2_] were also monitored. Plants were grown in hydroponic conditions, either in the presence (P+) or in the absence (P-) of the macronutrient P; plants were also either inoculated or not inoculated with *Enterobacter* sp. strain 15S. In addition, considering the strong P solubilization ability featured by *Enterobacter* sp. strain 15S, a set of plants were also fed an insoluble inorganic Pi source [Ca_3_(PO_4_)_2_] and monitored for their growth and mineral nutrient accumulation. After a cultivation period of 21 days, the plants were sampled and assessed for their growth, the concentration of mineral elements in their tissues, biological activities of their roots (i.e., release of exudates), and the molecular modulation of Pi transporters at the root level.

## Materials and Methods

### Experimental Design and Plant Growth Conditions

**Figure 1:**
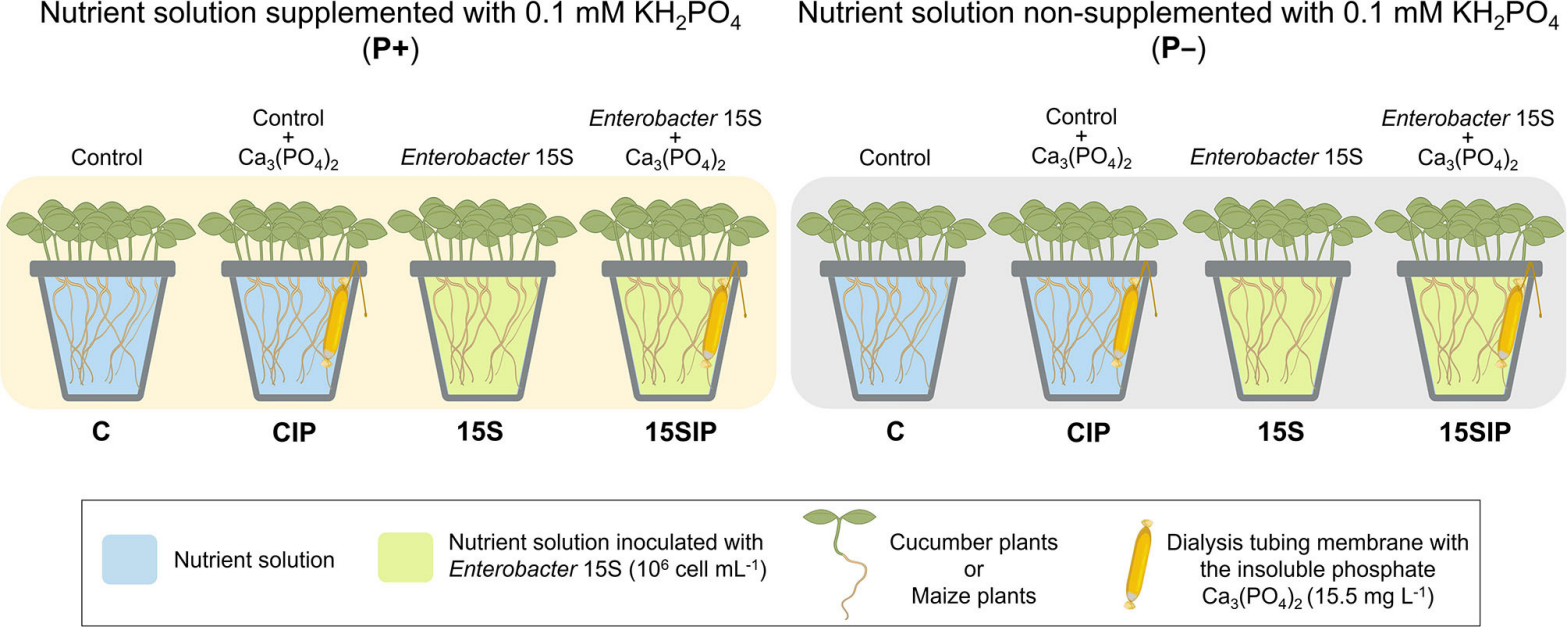
The experimental design of the present study. Seedlings of cucumber or maize were grown on nutrient solutions (NSs) supplemented (P+) or non-supplemented (P–) with 0.1 of mM KH_2_PO_4_. The bacterial biostimulant with *Enterobacter* 15S was used to inoculate the hydroponic NS to a final concentration of 10^6^ cell ml^−1^ (15S) and the NS in pots containing the insoluble phosphate Ca_3_(PO_4_)_2_ (15SIP). Two uninoculated controls, with the insoluble phosphate Ca_3_(PO_4_)_2_ (CIP) or without it (C), were also evaluated (Refer text in detail). The dialysis membrane used was a MEMBRA-CEL MD34 14 × 100, MWCO: 14000, Pore size: 50Å (Viskase Companies Inc., Willowbrook, IL, USA).

Cucumber (*Cucumis sativus* L. cv. Chinese Long) and maize (*Z. mays* L. – hybrid P0423, Pioneer Hi-Bred Italia S.r.l) plants were hydroponically grown under controlled environmental conditions in a climatic chamber with a 14/10-h light/dark period, 24/19°C, 250 μmol m^−2^ s^−1^ light intensity, and 70% relative humidity. Seeds were germinated for 5 days in the dark at 22°C on filter paper moistened with 0.5 mM CaSO_4_. Five-day-old seedlings were then transferred into 2-L plastic pots filled with 1.5 L of a full-strength nutrient solution (NS), either not supplemented (P-) or P-supplemented (P+) using the soluble phosphate 0.1 mM KH_2_PO_4_. The NS had the following composition: 2 mM Ca (NO_3_)_2_, 0.5 mM MgSO_4_, 0.7 mM K_2_SO_4_, 0.1 mM KCl, 10 μM H_3_BO_3_, 0.5 μM MnSO_4_, 0.2 μM CuSO_4_, 0.5 μM ZnSO_4_, 0.01 μM (NH_4_)_6_Mo_7_O_24_, and 80 μM Fe-EDTA. The solution was continuously aerated and changed two times per week. After 7 days of hydroponic culture, plants were set in a 2 × 4 factorial experimental design completely randomized with two P conditions (P+ and P–) and two biostimulant treatments with the PGPB *Enterobacter* 15S, with or without insoluble Ca_3_(PO_4_)_2_, provided with a dialysis tube (named 15S and 15SIP, respectively), plus two inoculated controls (named C and CIP, respectively) (Figure 1). Three biological replicates, with 10 (cucumber) or eight (maize) seedlings per replicate, were performed for each treatment. After 21 days of cultivation, the plants were harvested and assessed as described below.

### Bacterial Biostimulant

*Enterobacter* sp. 15S strain (KX884932.1) was originally isolated from bulk soil under conventional horticultural management in the Laboratory of Molecular Biochemistry of the State University of Londrina (Paraná, Brazil) (Zuluaga et al., 2020). The bacterial strain was grown in Luria-Bertani (LB) medium (10 g L^−1^ tryptone, 5 g L^−1^ yeast extract, and 10 g L^−1^ NaCl) under orbital shaking at 180 rpm, 28°C, for 24 h. After that period, the cells were harvested, washed three times, and resuspended in a sterile saline solution (0.85% w/v NaCl). Bacterial biostimulant suspension was used to inoculate the hydroponic NS to a final concentration of 10^6^ cell ml^−1^ (Figure 1). Control treatments were treated with the same amount of sterile saline solution.

### Plant Analysis

#### Morpho-Physiological Parameters

##### SPAD Values

After 15 days of hydroponic culture, the plants were harvested and the changes in chlorophyll concentration of the youngest fully expanded leaves were determined using a portable chlorophyll meter (SPAD-502, Minolta, Osaka, Japan) and presented as soil-plant analysis development (SPAD) units. Measurements were performed on three plants per pot of each of the three biological replicates and at least two readings per plant were taken and averaged.

##### Plant Biomass

At harvest, three biological replicates of the shoots and roots were separated and dried at 65°C until constant weight. The dry weight (DW) of the roots and shoots was recorded.

##### Root Morphological Characteristics

For the analysis of root morphology, fresh roots were scanned using a root scanner system (EPSON Perfection V800, Regent Instruments Inc., Quebec, Canada) and data were then analyzed with the WinRHIZO software (EPSON 1680, WinRHIZO Pro2003b) to determine the root characteristics, including length, surface area, diameter, volume, and the number of tips. All determinations were made in triplicates.

### Element Analysis

The plant tissue of the shoots and roots were dried at 65°C and acid digested with 68% ultrapure HNO_3_ (Carlo Erba, Milano, Italy) in a single reaction chamber microwave digestion system (UltraWAVE, Milestone, Shelton, CT, USA). Concentrations of macro and micronutrients were determined using an inductively coupled plasma–optical emission spectrometer (ICP-OES Spectro CirosCCD, Spectro, Germany). Element quantification was carried out using certified multi-element standards (CPI International, https://cpiinternational.com). Tomato leaves (SRM 1573a) and spinach leaves (SRM 1547) were used as the external certified reference materials. Determinations were carried out in triplicates.

### Root Exudation of Phenolics and Flavonoids

Root exudates were collected at the end of the hydroponic cultivation period. One plant of each biological replicate was removed from the NS and their roots were washed with distilled water. Plants were then transferred separately into smaller pots containing 20 ml of H_2_O MQ (18.2 MΩ cm^3^) as a trap solution. Pots were covered with aluminum foil to keep the roots in the dark, with the trap solution being continuously aerated. Root exudates were collected after 24 h, filtered at 0.45 μm, frozen at −80°C, freeze-dried, and resuspended in methanol 60%. The total phenolic content in the root exudates was determined using the Folin-Ciocalteau method (Folin and Ciocalteau, 1927) and was expressed as the nmol equivalent of gallic acid per gram of root fresh weight (RFW). Total flavonoid content was measured with the aluminum chloride colorimetric method (Miliauskas et al., 2004) and expressed as the nmol equivalent of rutin per gram of root fresh weight.

### Gene Expression Analysis

Root tissues from two plants of each biological replicate were collected. The harvested roots were frozen in liquid N and stored at −80°C until use. Total RNA was extracted from frozen roots using the Spectrum Plant Total RNA Kit (Sigma-Aldrich, St. Louis, MO, USA) according to the instructions of the manufacturer. The total RNA (1 μg) was treated with 10 U of DNAse RQ1 and used for cDNA synthesis using the ImProm-II Reverse Transcription System (Promega, Madison, WI, USA) and oligo (dT)_15_ primer as per the recommendations of the manufacturer. The cDNA obtained was used as a template for the quantitative real-time reverse transcription PCR (qRT-PCR) performed using the SsoFast EvaGreen Supermix (Bio-Rad, Hercules, CA, USA) and the Bio-Rad iCycler MyiQ real-time PCR system (Bio-Rad). Gene-specific primers were designed for the target genes and the housekeeping gene, the elongation factor 1α (Supplementary Table 1). Experiments were carried out in triplicates with the following conditions: 5 min at 95°C, followed by 40 cycles at 95°C for 30 s, and 55°C for 30 s, as described previously (Pii et al., 2016, 2019). The amplification efficiency was calculated from raw data using the LinRegPCR software (Ramakers et al., 2003). For each transcript, the mean normalized expression value (MNE; Simon, 2003) was calculated using the housekeeping transcript and the relative expression ratio values were calculated by the 2^−Δ*ΔCt*^ method according to Livak and Schmittgen (2001).

### Data Analysis

All experimental data for both plant species (*C. sativus* and *Z. mays*) were statistically subjected to two-way ANOVA using the software IBM SPSS Statistics 20. The mean values were separated according to Tukey's HSD test with *p* < 0.05 and *P* levels effects were compared using the *t*-test. A heatmap summarizing the morpho-physiological parameters, ionomic analysis, and root exudation responses of both plant species to the biostimulant treatments and P fertilization levels were also generated using the R software (https://www.r-project.org, performing “pheatmap,” “ggplot2,” and “RColorBrewer” packages). Hierarchical clustering based on standardized data was performed with Complete linkage and Manhattan distance was used as the similarity measure.

## Results

### Plant Growth and Morpho-Physiological Parameters

**Figure 2:**
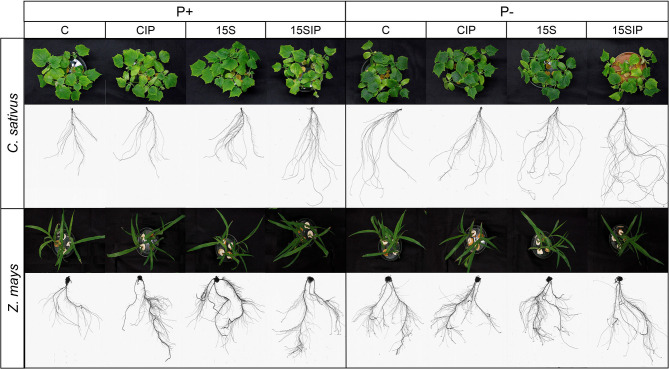
Shoot and root apparatus of cucumber (*C. sativu*s) and maize (*Z. mays*) plants in response to a factorial experiment with two P fertilization levels (P+, NS supplemented with 0.1 mM of KH_2_PO_4_; P–, NS non-supplemented with KH_2_PO_4_) and four biostimulant treatments [C, uninoculated control; CIP, uninoculated control with the insoluble phosphate Ca_3_(PO_4_)_2_; 15S, inoculated with the PGPB *Enterobacter* 15S; 15SIP, inoculated with the PGPB *Enterobacter* 15S plus the insoluble phosphate Ca_3_(PO_4_)_2_].

At the end of the growing period, P deficiency in plants was visually and more identified in cucumber plants when compared with maize plants, since they showed typical symptoms like the dark green color of the leaves and higher expansion of the root system (Figure 2). However, for both plant species, the ANOVA results showed that most of the measured morpho-physiological parameters were significantly affected by P fertilization levels (P) and biostimulant treatments (I), except for the root diameter, which was not affected by the biostimulant treatments (Supplementary Table 2). The P × I interaction effects were also significant for both plant species, influencing most of the parameters (Supplementary Table 2). This interaction was better noted when comparing the mean values, as shown in Table 1. For instance, in cucumber plants, the SPAD index values were significantly higher in P-deficient plants (P–) when compared with P-fed plants (P+). As expected, P-deficient cucumber plants appeared darker in color (Figure 2) with the exception of the 15SIP treatment. In the latter, the plants did not exhibit darker green leaves with average values not different from those measured in plants grown in P-full condition (P+: 15SIP) (Table 1). Similarly, in maize plants, the highest SPAD index values were recorded under P deficiency with no visible symptoms of pigmentation on the leaves. However, in both inoculation treatments, maize plants presented lower SPAD values, which were no different from those recorded in plants grown under full P (Table 1). It is interesting to note that, for P-fed maize plants, the effect of the bacterial inoculation on the levels of chlorophyll contents was not significant when compared to the control. Nevertheless, a more evident effect was noted in P-deficient maize plants, suggesting the involvement of the bacteria in the amelioration of photosynthetic rates during P starvation.

**Table 1:** Mean values for the morpho-physiological parameters in cucumber and maize grown hydroponically under different P-fertilization levels and different inoculation treatments with *Enterobacter* 15S.

**Parameters^a^**	**P level^b^**	**Cucumber**					**Maize**				
		**Biostimulant treatments^c^**					**Biostimulant treatments^c^**				
		**C**	**CIP**	**15S**	**15SIP**	**P level effect**	**C**	**CIP**	**15S**	**15SIP**	**P level effect**
SPAD	P+	29.97 Ba	29.93 Ba	29.17 Bab	28.80 b	29.47 B	29.47 B	31.20 B	31.40	30.97	30.76 B
	P-	43.63 Aa	41.07 Aa	43.57 Aa	30.20 b	39.62 A	38.03 Aa	38.07 Aa	32.90 b	30.63 b	34.91 A
Treatment effect		36.80 a	35.50 a	36.37 a	29.50 b		33.75 ab	34.63 a	32.15 bc	30.80 c	
Length	P+	194.15 Bb	218.40 Bab	232.52 Bab	285.17 Ba	232.56 B	229.42 Bb	522.94 a	541.27 a	456.97 Ba	437.65
	P-	424.07 Aa	305.37 Ac	330.57 Abc	374.56 Aab	358.64 A	503.23 A	500.49	519.83	522.16 A	511.43
Treatment effect		309.11 ab	261.88 c	281.54 bc	329.87 a		366.33 b	511.72 a	530.55 a	489.57 a	
Surface area	P+	17.62 Bb	19.70 Bb	22.71 Bab	28.21 Ba	22.06 B	24.53 Bd	61.88 Aa	54.94 b	47.76 c	47.28
	P-	41.26 Aa	29.08 Ab	31.81 Ab	40.16 Aa	35.58 A	46.80 Ab	47.55 Bb	53.01 a	50.40 ab	49.44
Treatment effect		29.44 b	24.39 c	27.26 bc	34.18 a		35.67 c	54.72 a	53.98 a	49.08 b	
Diameter	P+	0.29 B	0.30	0.30	0.30	0.30 B	0.32	0.34 A	0.33	0.33	0.33 A
	P-	0.34 Aa	0.30 b	0.32 ab	0.32 ab	0.32 A	0.32	0.31 B	0.33	0.31	0.32 B
Treatment effect		0.32	0.30	0.31	0.31		0.32	0.33	0.33	0.32	
Volume	P+	0.13 Bb	0.15 Bb	0.18 Bab	0.22 Ba	0.17 B	0.24 Bc	0.54 Aa	0.49 ab	0.39 b	0.42
	P-	0.26 Aab	0.22 Ab	0.24 Aab	0.32 Aa	0.26 A	0.37 A	0.36 B	0.43	0.39	0.39
Treatment effect		0.20 b	0.18 b	0.21 b	0.27 a		0.31 c	0.45 a	0.46 a	0.39 b	
Tips	P+	231.00 Bab	214.67 Bab	198.00 Bb	306.67 Ba	237.58 B	374.67 Bb	613.67 a	691.33 a	704.00 a	595.92
	P-	445.67 Aa	304.67 Ab	350.00 Aab	405.33 Aab	376.42 A	672.00 Aab	574.00 b	745.33 a	669.33 ab	665.17
Treatment effect		338.33 a	259.67 b	274.00 b	356.00 a		523.33 b	593.83 b	718.33 a	686.67 a	
RDW	P+	15.03 ab	10.50 Bc	17.00 a	11.90 Bbc	13.61 B	25.43 Aa	30.97 Aa	27.63 Aa	14.97 b	24.75
	P-	14.27	15.93 A	17.83	15.70 A	15.93 A	19.83 B	25.17 B	20.33 B	19.17	21.13
Treatment effect		14.65 b	13.22 b	17.42 a	13.8 b		22.63 b	28.07 a	23.98 ab	17.07 c	
SDW	P+	86.97 Aa	89.13 a	82.67 Aa	61.70 b	80.12	146.70 Aa	133.00 Aa	95.93 Ab	100.33 b	118.99 A
	P-	66.37 Bab	85.53 a	66.17 Bab	62.27 b	70.08	94.27 Ba	71.10 Bb	59.43 Bb	93.3 a	79.53 B
Treatment effect		76.67 ab	87.33 a	74.42 ab	61.98 b		120.48 a	102.05 b	77.68 c	96.82 b	
R/S ratio	P+	0.17 Ba	0.12 Bb	0.21 Ba	0.19 Ba	0.17 B	0.17 b	0.23 Ba	0.29 Ba	0.15 Bb	0.21 B
	P-	0.22 Ab	0.19 Ab	0.27 Aa	0.25 Aa	0.23 A	0.21 b	0.35 Aa	0.34 Aa	0.21 Ab	0.28 A
Treatment effect		0.20 ab	0.15 b	0.24 a	0.22 a		0.19 b	0.29 a	0.31 a	0.18 b	

*Phosphorus level effects were compared using t-tests. Differences between means were determined using Tukey's HSD test. Significant differences (p < 0.05) according to Tukey's test are indicated by different capital letters when comparing contrasts in columns and different lowercase letters when comparing contrasts in rows. No significant differences are indicated by omitting notation letters*.

a *soil-plant analysis development (SPAD), SPAD units; Length, total root length (cm); Surf. area, root surface area (cm^2^); Diameter, root diameter (cm); Volume, total root volume (cm^3^); Tips, Number of root tips; RDW, root dry weight (mg); SDW, shoot dry weight (mg); R/S, root-to-shoot ratio*.

b *P levels: P+ [nutrient solution (NS) supplemented with 0.1 mM of KH_2_PO_4_]; P– (NS non-supplemented with KH_2_PO_4_)*.

c *Biostimulant treatments: C, uninoculated control; CIP, uninoculated control with the insoluble phosphate Ca_3_(PO_4_)_2_; 15S, inoculated treatment with the PGPB Enterobacter 15S; 15SIP, inoculated treatment with the PGPB Enterobacter 15S plus the insoluble phosphate Ca_3_(PO_4_)_2_*.

In both plant species, the biomass and the architecture of the root systems were also affected by both P levels and inoculation treatments. For instance, in cucumber, the mean values recorded for root DW, total root length, surface area, volume, tips, and the root/shoot (R/S) ratio were significantly higher under P deficiency when compared with plants grown with full P; on the contrary, the shoot DW was higher under complete P (Table 1). Furthermore, when inoculated with *Enterobacter* 15S, P-deficient cucumber plants showed a significant decrease in the root length and surface area with respect to the uninoculated control, whereas supplementation with the insoluble Ca_3_(PO_4_)_2_ (treatment P–: 15SIP) produced a significant increase in these parameters, albeit not significant when compared to the control. Additionally, root and shoot biomass were unaffected by the inoculation, whereas the R/S ratio was highly increased in both inoculant treatments (Table 1). A different response to the inoculation was observed in P+ cucumber plants. In particular, the length, surface area, and volume of roots were significantly increased with the use of *Enterobacter* 15S alone or in combination with the insoluble phosphate (P+: 15S and 15SIP). However, the root and shoot biomass of the cucumber plants inoculated with *Enterobacter* alone were not significantly different from control plants; instead, its combination with Ca_3_(PO_4_)_2_ produced a reduction in plant biomass accumulation, while the R/S ratio was not affected.

In contrast with cucumber, P fertilization levels did not affect the root architecture of maize plants. Nonetheless, the root and shoot biomass were significantly higher under the P-full condition, while the R/S ratio increased in P-deficient plants (Table 1). Moreover, P– maize plants inoculated with *Enterobacter* 15S did not show significant changes in length, diameter, volume, and root biomass as compared to control plants. On the other hand, the surface area and root tips were significantly increased in plants treated with the bacteria alone (P–: 15S), albeit a reduction of 37% in the shoot biomass was observed. In P+ maize plants, the inoculation with the *Enterobacter* alone (P+: 15S) significantly increased the architecture of the root system as compared to control plants, albeit the root biomass was not affected; on the other hand, a decrease in the shoot biomass was recorded.

### Evaluation of Element Composition in the Roots and Shoots of Cucumber and Maize

**Figure 3:**
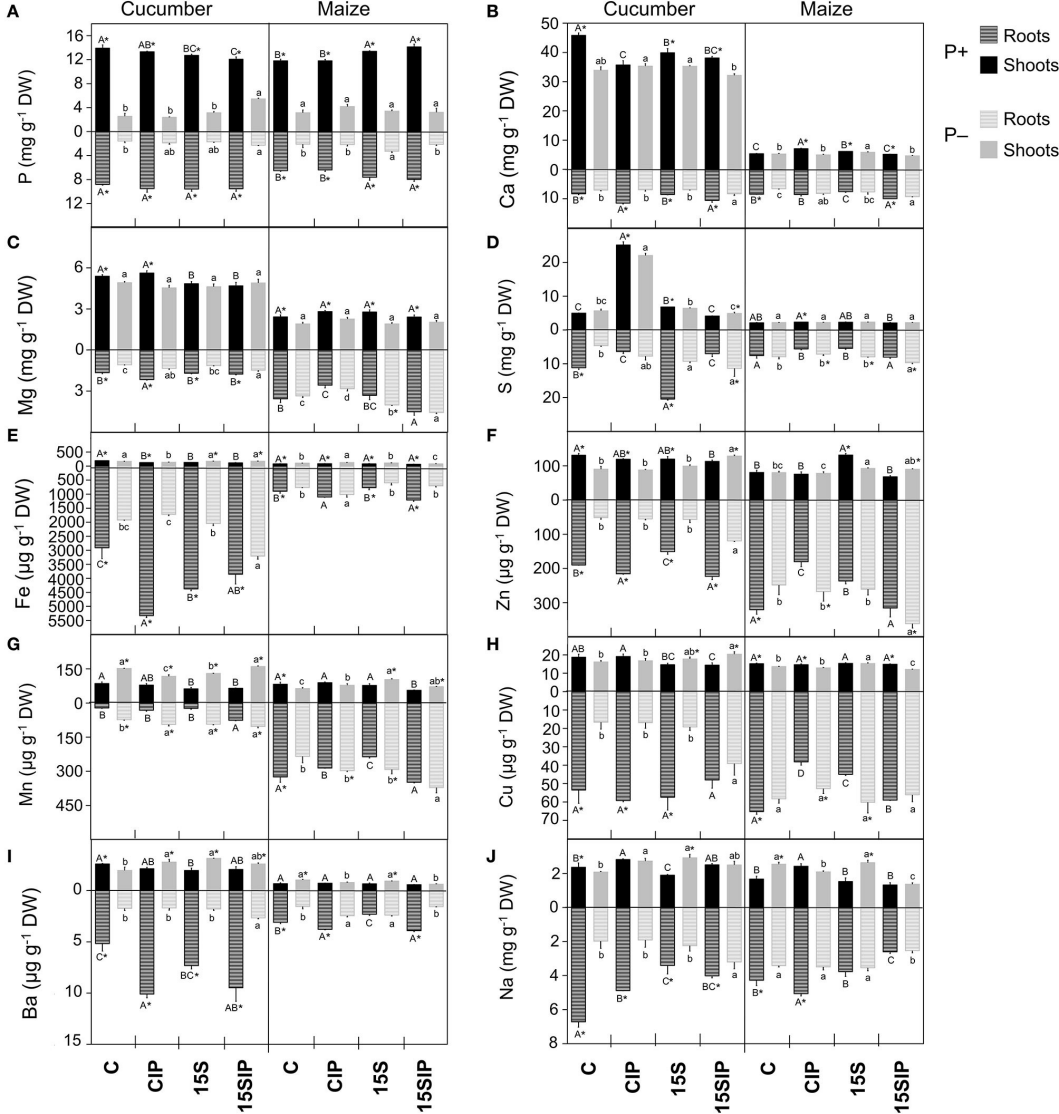
Ionomic profile of cucumber and maize plants tissues. **(A)** Phosphorus, P; **(B)** Calcium, Ca; **(C)** Magnesium, Mg; **(D)** Sulfur, S; **(E)** Iron, Fe; **(F)** Zinc, Zn; **(G)** Manganese, Mn; **(H)** Copper, Cu; **(I)** Barium, Ba; **(J)** Sodium, Na concentrations in roots and shoots of cucumber and maize grown hydroponically under different P-fertilization levels (P+ and P–) and different biostimulant treatments with *Enterobacter* 15S (C, CIP, 15S, 15SIP). Values are means ± SE; *n* = 3. Uppercase letters compare treatments under P+, and lowercase letters compare treatments under P–. An asterisk is present when there is a difference in the same treatment between P+ and P–. Equal letters correspond to average values that do not differ according to Tukey's test (*p* < 0.05).

A suite of 10 mineral elements, among which were macronutrients (P, calcium - Ca, magnesium – Mg, and sulfur - S), micronutrients (iron - Fe, zinc - Zn, manganese - Mn, and copper - Cu), and beneficial nonessential elements (barium - Ba and sodium - Na), were analyzed in the roots and shoots of both plant species (Figure 3 and Supplementary Table 3). In cucumber, regardless of P fertilization and PGPB inoculation, foliar concentrations of P, Ca, Mg, and Mn exceeded those in the roots, while Fe and Cu concentrations were higher in the roots. On the other hand, the concentrations of Ba, Zn, S, and Na were dependent on both P fertilization and PGPB inoculation. For instance, the elements mentioned were higher in roots of P+ cucumber plants as compared to shoots but displayed higher concentrations in the shoots of P– plants (Figure 3). In maize, the concentration of all the mineral elements was higher in roots, regardless of the P levels and inoculant treatments, except for P concentration, which was higher in aboveground tissues.

Nevertheless, P fertilization level and biostimulant treatments caused the imbalanced nutrient distribution in the roots and shoots of both plant species. As expected, P deficiency led to a decreased P concentration in the roots and shoots of both plants (Figure 3A). However, in cucumber, the inoculation with *Enterobacter* 15S in combination with Ca_3_(PO4)_2_ (treatment P–: 15SIP) significantly increased the content of P in the roots and shoots by about 31 and 53%, respectively, as compared to the P– uninoculated samples (P–: C). In contrast, in maize, P-deficient plants inoculated with the bacteria alone (P–: 15S) led to the enhancement of P concentration in the roots (+36%) as compared to uninoculated plants (P–: C); however, P concentration in the shoots was not affected (Supplementary Table 3). In the P+ condition, the P concentration values in the roots of the cucumber plants were unaffected by the treatments imposed, whereas inoculation with PGPB induced a slight decrease of this mineral in the shoots. On the contrary, in maize plants, inoculation with *Enterobacter* caused a higher accumulation of P in both the roots and shoots (Figure 3A).

The accumulation of Ca and Mg in the roots and shoots of both cucumber and maize grown in P deficiency was decreased when compared to plants grown in P+ conditions (Figures 3B,C). An exception to this trend is represented by maize roots, in which the inoculation with *Enterobacter* 15S (treatment P–: 15S) induced a higher accumulation of Mg compared to P+ plants. On the contrary, in P+ cucumber plants, treatments with the inoculants caused a significant decrease in Mg concentration (Supplementary Table 3). Calcium concentration was, in both P+ and P– plants, significantly increased in the roots of both 15SIP cucumber and maize, although the same treatment induced the opposite effect in cucumber shoots and no effects in maize shoot when compared to the control. Additionally, the treatment with the bacterium (i.e., 15S) resulted in the highest content of Ca in the shoots of all the maize plants.

Under P deficiency, S concentration in the roots of cucumber was increased by the 15SIP treatment as compared to P+ conditions, whereas, in the case of maize, only inoculated P-deficient plants (i.e., P–: 15S and 15SIP) showed higher S content in the roots with respect to the plants grown under P+ conditions (Figure 3D). The inoculation with *Enterobacter* significantly increased the concentration of S by more than 45% in the roots of both P-full and P-deficient cucumber plants compared to the control alone, while the highest content in shoots was observed in control plants combined with Ca_3_(PO_4_)_2_ (treatment CIP). In contrast, the different treatments did not affect the accumulation of S in maize shoots.

Concerning the micronutrients (Figures 3E–H) and non-essential elements (Figures 3I,J), the P deprivation in the NS led to a decreasing trend in all the elements in the roots of cucumber plants, except for Mn, but caused an increase in the concentration of Fe, Mn, and Ba in the aboveground tissues. In maize plants, P deficiency induced the increase in Fe and Ba concentration at the shoot level, as well Zn and Mn in the roots. Furthermore, under P deficiency conditions, the inoculation of cucumber with *Enterobacter* 15S in combination with the insoluble phosphate (P–: 15SIP) had a significant promoting effect on the accumulation of all micronutrients in the roots when compared to the uninoculated control, whereas the same treatment had no significant effects on Fe and Mn concentration in the shoots. However, under P-sufficient conditions, a decrease in the content of all micronutrients in the shoots of cucumber was associated with *Enterobacter* 15S inoculation. Besides, the supplementation with Ca_3_(PO_4_)_2_ (treatment P+: CIP) induced the highest Fe, Zn, and Ba concentrations in cucumber roots (Figure 3 and Supplementary Table 3). On the other hand, P-deficient maize plants treated with the bacterium plus Ca_3_(PO_4_)_2_ (treatment P–: 15SIP) showed a significant increase in the concentration of Mn and Zn in the roots and a decrease in that of Na; no changes were observed for Fe and Cu. When the bacterial inoculant was used alone, the highest concentrations of Zn, Mn, and Cu were detected in the leaves of maize as compared with uninoculated plants. In contrast, the inoculation treatments did not induce remarkable changes in the accumulation of micronutrients in the roots and shoots of maize plants grown under the full-P NS (Figures 3E,J). Additionally, under P+ conditions, the inoculated maize and cucumber plants showed a decreased content of Na in the roots when compared to the uninoculated plants (Figure 3J, treatments P+: 15S and 15SIP).

### Release of Phenolics and Flavonoids

**Figure 4:**
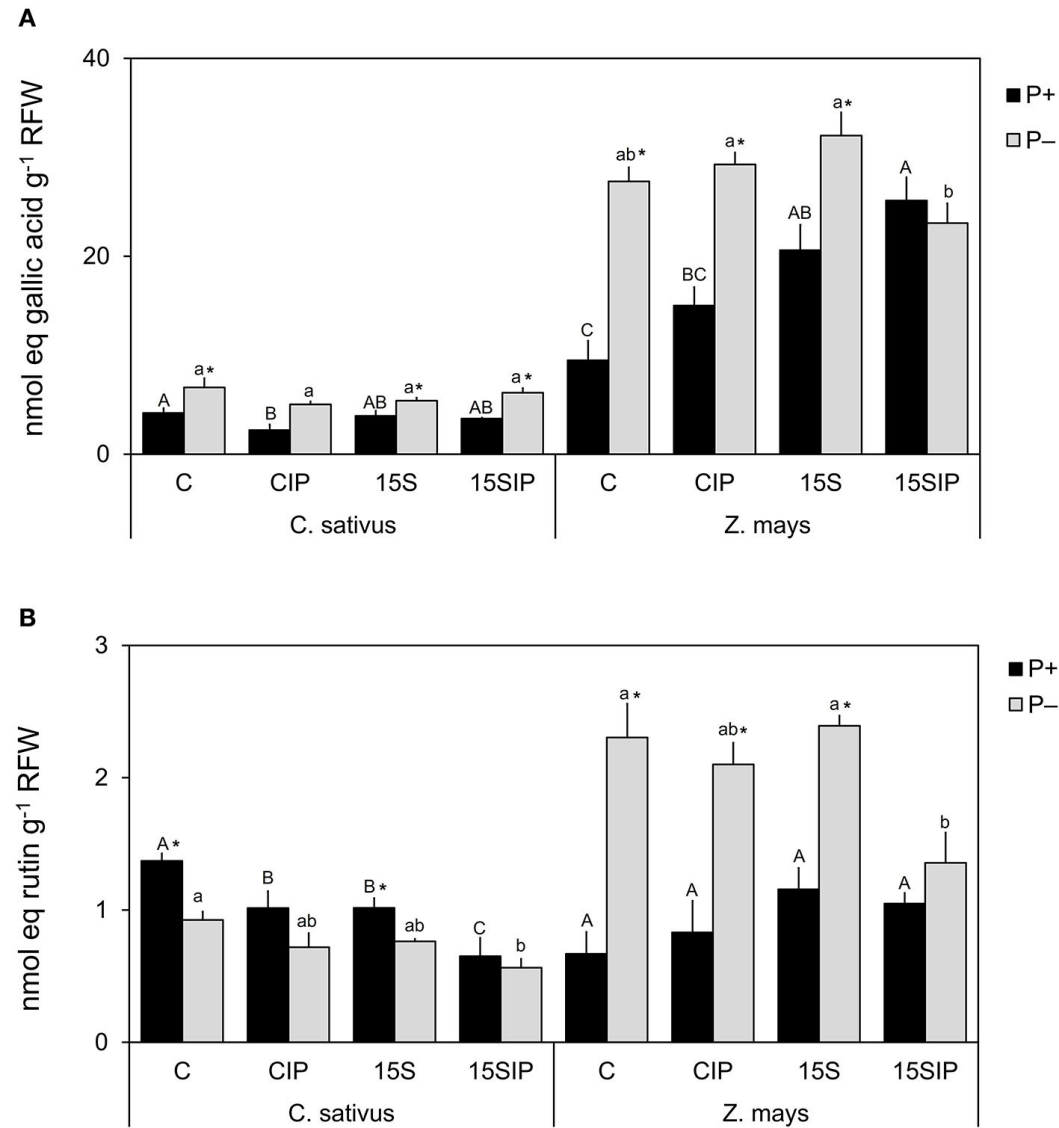
Total phenolic compounds content **(A)** and total flavonoid content **(B)** determined in the root exudates of cucumber and maize collected at the end of the cultivation period. Values are means ± SE; *n* = 3. Capital letters compare treatments under P+, and lowercase letters compare treatments under P–. An asterisk is present when there is a difference in the same treatment between P+ and P–. Equal letters correspond to average values that do not differ according to Tukey's test (*p* < 0.05). Two-way ANOVA results: Phenolic compounds: cucumber, P (*p* < 0.001), I (*p* < 0.01), P × I (NS); maize, P (*p* < 0.001), I (*p* < 0.001), P × I (*p* < 0.001). Flavonoids: cucumber, P (*p* < 0.001), I (*p* < 0.001), P × I (NS); maize, P (*p* < 0.01), I (NS), P × I (*p* < 0.01).

The root exudate concentration released by both plant species was determined spectrophotometrically in terms of phenolic compounds and flavonoids. Figure 4A shows the total content of phenolic compounds released by the cucumber and maize plants in the different conditions considered in the study. In cucumber, the amount of phenolics was higher in P-deficient conditions (ranging from 5 to 7 nmol g^−1^ RFW) compared to P+ plants (ranging from 2 to 4 nmol g^−1^ RFW). Any statistically significant difference was recorded between the different treatments. Under the P+ conditions, the content of phenolics was significantly higher in the control treatment, while inoculation with *Enterobacter* 15S induced a smaller release of phenolic compounds (Figure 4A). In maize plants, the root exudation of phenolics was higher as compared to cucumber. In the P+ condition, inoculation with the bacterium (P+: 15S and 15SIP treatments) induced a higher exudation of phenolics compared to the uninoculated controls. On the other hand, the release of phenolics by maize plants grown in P deficiency was significantly higher (23–32 nmol g^−1^ RFW) than that observed in P+ conditions (9–26 nmol g^−1^ RFW), except for the treatment P–: 15SIP. In the latter, the bacterial inoculation and supplementation with Ca_3_(PO4)_2_ induced a decrease in the exuded phenolics, presenting levels significantly lower than that measured in control plants (Figure 4A).

The root exudation of flavonoids by both plant species is shown in Figure 4B. In cucumber, the total flavonoid content was significantly reduced by the bacterial inoculation in both P fertilization levels. However, in P+ conditions, a significantly higher production was observed for C and 15S treatments (1 and 1.4 nmol g^−1^ RFW, respectively), compared with the same treatments under P deficiency (0.8 and.9 nmol g^−1^ RFW, respectively); any remarkable effects were observed for CIP and 15SIP in both P+ and P– conditions (Figure 4B). On the other hand, P deficiency induced a higher exudation of flavonoids in maize (ranging from 2.1 to 2.4 nmol g^−1^ RFW) with respect to P+ plants (ranging from 0.7 to 1.2 nmol g^−1^ RFW), except for the 15SIP treatment. No significant effects were observed within P+ plants subjected to different treatments.

### Phosphate Transporters Gene Expression Analysis

In plants, Pi transporter genes belonging to the *Pht1* family are strongly expressed in roots and play important roles in the transmembrane transport of Pi for its acquisition and allocation within the plants and the single cell. Most of these genes have been reported to be highly induced by P deficiency (Wang et al., 2018). In cucumber, six genes involved in the phosphate uptake have been identified, namely, *CsPT1.3, CsPT1.4, CsPT1.7, CsPT1.9, CsPT1.11*, and *Cucsa383630.1* (Naureen et al., 2018; Feil et al., 2020). However, under the conditions, the expression of *CsPT1.7* and *CsPT1.11* was not detectable under P starvation (data not shown). In maize, 13 *PhT1s* genes have been identified (Liu et al., 2016), but in this work, six of these genes were reported by Nagy et al. (2006), namely, *ZmPT1, ZmPT2, ZmPT3, ZmPT4, ZmPT5*, and *ZmPT6* were analyzed. Nonetheless, four genes (*ZmPT2, ZmPT3, ZmPT4*, and *ZmPT6*) were poorly induced by P deficiency in our experimental conditions (data not shown).

**Figure 5:**
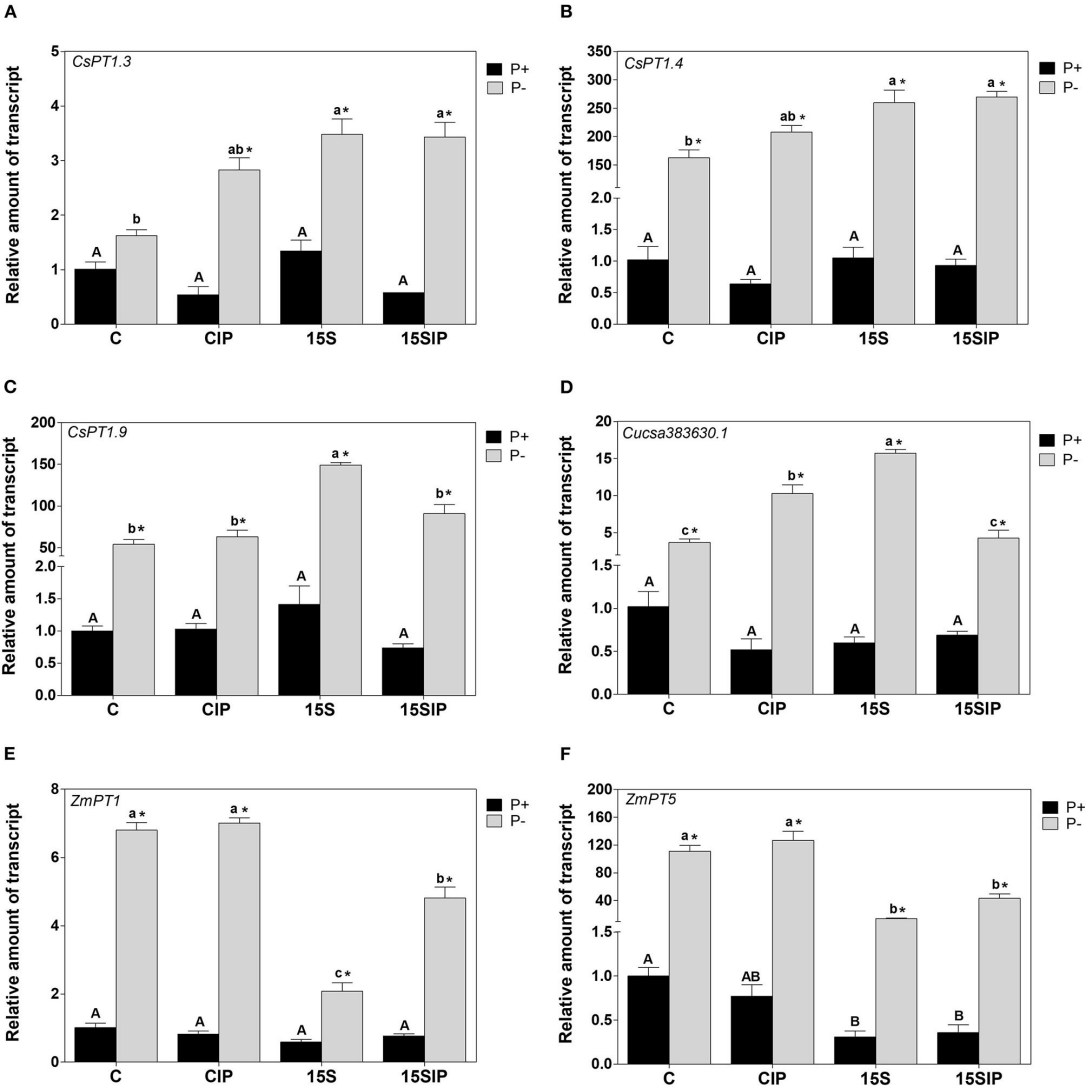
Gene expression analysis of **(A)** *CsPT1.3*, **(B)** *CsPT1.4*, **(C)** *CsPT1.9*, and **(D)** *Cusca383630.1* in the roots of the cucumbers, and **(E)** *ZmPT1* and **(F)** *ZmPT5* in the roots of maize grown hydroponically under different P-fertilization levels (P+ and P–) and different biostimulant treatments with *Enterobacter* 15S (C, CIP, 15S, 15SIP). The expression level of each gene was normalized to the expression level of the elongation factor isoform 1-alpha (EF-1α). The relative expression ratios were calculated using the uninoculated control under complete P (P+: C) as a calibrator sample. Values are means ± SE; *n* = 3. Uppercase letters compare treatments under P+ and lowercase letters compare treatments under P–. An asterisk is present when there is a difference in the same treatment between P+ and P–. Equal letters correspond to average values that do not differ according to Tukey's test (*p* < 0.05).

Figure 5 shows the relative expression levels of the *CsPTs* and *ZmPTs* genes under P deficiency. Results indicate that, in both plants, all the analyzed genes were expressed under the P+ condition but were highly upregulated in P-deficiency. For instance, in cucumber, *CsPT1.4* was highly induced in P- (>160-fold up to 270-fold), followed by *CsPT1.9* (>50-fold up to 150-fold) and *Cucsa383630.1* (>4-fold up to 15-fold), while *CsPT1.3* was less expressed (>2-fold) when compared to control plants under complete P fertilization. Additionally, in cucumber plants grown under a P-full NS, the expression of all *CsPTs* genes was not significantly affected by the treatments (Figure 5). On the other hand, inoculation with *Enterobacter* 15S induced transcriptional changes for all *CsPTs* genes under P starvation. For instance, *CsPT1.3* and *CsPT1.4* were induced by both inoculant treatments (P–: 15S and 15SIP) in comparison to uninoculated plants (P–: C and CIP). On the contrary, inoculation with the bacterium (P-: 15S) induced a significantly higher expression of the *CsPT1.9* and *Cucsa383630.1* genes as compared with the other three treatments.

In maize plants grown under P deficiency, *ZmPT5* was expressed more (>14-fold up to 127-fold) than *ZmPT1* (>2-fold up to 7-fold), compared with P-sufficient control plants. However, the inoculation induced different relative transcript levels in both genes. For instance, in maize plants grown in either P-full or P-deprived NSs, inoculation with *Enterobacter* 15S downregulated the expression of *ZmPT5* in roots when compared to uninoculated control plants under the same conditions (Figure 5). Nonetheless, the relative transcript levels of *ZmPT1* were significantly lower in P-deficient plants inoculated with the bacterium alone (P–: 15S), while the inoculant treatment supplemented with the insoluble phosphate (P–: 15SIP) induced the expression of a higher amount of transcript, yet not much higher than the uninoculated controls (Figure 5). No significant differences were observed in the expression of *ZmPT1* in maize plants under P-full conditions.

## Discussion

### Plant Growth and Morpho-Physiological Responses to Bacterial Biostimulant Inoculation and/or P Supply

The inoculation with PGPB has been shown to improve plant growth in both favorable and unfavorable conditions, including low-nutrient availability (Oleńska et al., 2020). In the specific case of P) shortage, it has been broadly described that physiological, morphological, and molecular changes are induced in plants as a specific response to this nutritional stress (López-Arredondo et al., 2014; Elanchezhian et al., 2015). Results reported in this study show that both P availability in the NS and the bacterial biostimulant *Enterobacter* 15S were able to influence the plant biomass accumulation and the development of morpho-physiological traits in cucumber and maize plants, although to a different extent. The cucumber plants exhibited a more remarkable response to P starvation, in particular, showing a more developed root system with respect to the maize plants. Indeed, the root system is very responsive to low-P availability in the growth medium; thus, in the majority of plant species, increasing root length root surface area, and enhancing the growth of root hairs are described as common strategies to explore larger soil surfaces and intercept nutrients (Ramaekers et al., 2010). Despite the root system architecture being different between monocots and dicots, the signaling pathways underpinning P-acquisition mechanisms are conserved (Shahzad and Amtmann, 2017). The low-P availability induces an increase in the R/S ratio that is generally ascribable to a limitation in shoot biomass accumulation, an increase in root production, or both (Campos et al., 2019). However, in our experimental conditions, the biomass allocations among the two plant species resulted quite differently. While the root biomass in maize plants was reduced under P starvation, it was significantly increased in the cucumbers. In this regard, it is worth mentioning that, from a nutrient availability perspective, plants have to balance the biomass allocation to leaves and/or roots to an extent that matches the physiological activities and functions performed by these organs (Poorter et al., 2012), including those related to plant responses to nutritional disorders.

The inoculation with the bacterial biostimulant *Enterobacter* 15S affected the root architecture and biomass of both plants differently depending on P supply. Overall, the data suggest that *Enterobacter* 15S led to the improved growth of the root system in both plant species grown in P-sufficient conditions. Under P deficiency, the bacterium in combination with Ca_3_(PO_4_)_2_ (P–: 15SIP) induced a remarkable effect on the whole cucumber plant, whilst the bacterium alone (P–: 15S) was more effective in improving the root system of maize plants. *Enterobacter* strains have been described to enhance the plant growth of different plant species, including monocot and dicot types (Naveed et al., 2013; Gupta et al., 2020; Ji et al., 2020). The biostimulant strain 15S used in this work was previously reported by Zuluaga et al. (2020) to have the ability to produce auxins and solubilize Pi. Bacteria-derived auxins are well-known for promoting morphological and physiological processes in plants, leading to increased growth of the root system in terms of root length and surface area; thus enhancing nutrient and water uptake (Hakim et al., 2021). Under stressful conditions (e.g., P deficiency), plants can stimulate bacteria to produce auxins (Kudoyarova et al., 2019). However, when produced in high concentrations, bacteria-derived auxins can inhibit root elongation in dicots, whereas monocots resulted in less sensitivity to such inhibition (Kudoyarova et al., 2019). In this context, it is also worth mentioning that a particular plant nutritional state (e.g., P sufficiency or P deficiency) may influence the interaction mechanisms between PGPB and host plants (Pii et al., 2016), thus explaining the contrasting results observed in this study for cucumber and maize plants. Moreover, it is important to highlight that, in a specific edaphic condition, PGPB may also display a differential preference for one particular plant species over another (Glick, 2005). The effects of this interaction rely on a set of adaptation mechanisms by both the inoculated bacteria and the host plant (Drogue et al., 2012).

Concerning chlorophyll content, a significant increase in both plant species grown under P starvation has been recorded, is particularly pronounced in cucumber leaves manifesting a particular dark green color. A similar trend has also been described in strawberry and apple plants (Valentinuzzi et al., 2015, 2019; Delaporte-Quintana et al., 2017). Moreover, a more severe or prolonged P deficiency may result in the accumulation of anthocyanins, consequently increasing the pigmentation of the newest leaves and chlorophyll concentrations (Veazie et al., 2020). However, the inoculation of P-deficient maize plants with *Enterobacter* 15S (i.e., 15S and 15SIP) reduced the levels of chlorophyll content to the same values of the inoculated P-sufficient plants. In contrast, in cucumber plants, only the treatment with the bacterium in combination with the insoluble phosphate (15SIP) was able to induce chlorophyll contents similar to those measured in P-sufficient plants. In this respect, Delaporte-Quintana et al. (2017) already reported that the SPAD values of P-deficient strawberry plants inoculated with *Gluconacetobacter diazotrophicus* PAL5 were similar to those obtained in plants grown under sufficient P, indicating the contribution of this strain to plant P nutrition and photosynthesis.

### Effects of Bacterial Biostimulant and P Supply on Mineral Nutrient Content

Several agronomic conditions such as physical-chemical properties of the growth substrate, fertilization regimes, and inoculation with PGPB have been described to induce changes in the elemental composition of crops, regardless of the clade considered (Pii et al., 2015a,b). In the present study, P deficiency led to a reduction of P content in the roots and shoots of both plants, while inoculation with the bacterial biostimulant with *Enterobacter* 15S induced a differential allocation of this element. For instance, in P-deficient cucumber plants, the inoculation in combination with Ca_3_(PO_4_)_2_ (P–: 15SIP) induced a higher allocation of P in roots and shoots, whereas maize plants inoculated with the bacterial biostimulant alone showed a higher content of P only in roots. Recent study showed that the inoculation of tomato plants grown under P-deficiency and salt stress conditions with different strains of *Arthrobacter* and *Bacillus* induced an increased P concentration in roots and shoots (Tchakounté et al., 2020) and the shoots of *Brachypodium* plants (Schillaci et al., 2021). On the contrary, the inoculation of wheat plants with beneficial bacteria led to a reduced P content in roots (Talboys et al., 2014). Interestingly, under P-sufficient conditions, *Enterobacter* 15S induced opposite effects on the P concentration in the shoots of the two plant species, showing a decrease in cucumber and an increase in maize plants. The results suggest that the mechanisms by which *Enterobacter* 15S improves P acquisition and its translocation within the plant can be dependent on both plant species and nutritional status. It was observed in cultivated monocots grown at different P levels that the inoculation with PGPB can increase the adaptation of these grasses to the low-P availability through an improvement in P-use efficiency, with the functionality of the P-uptake mechanism still not being affected (Pereira et al., 2020; Schillaci et al., 2021). Moreover, in dicots grown under different P states, it has been described that plant development stimulation *via* PGPB inoculation was not accompanied by an increase in nutrient uptake by the roots and the P content in shoots that, on the contrary, appeared to be rather decreased (Belimov et al., 2002).

Regarding micronutrients (i.e., Fe, Zn, Mn, and Cu) and non-essential beneficial elements (i.e., Ba and Na), inoculation with *Enterobacter* 15S promoted their accumulation at the root level only in cucumber plants supplemented with the insoluble phosphate grown under P-deficient conditions. The same trend was also observed at the shoot level, except for Fe and Mn. In maize plants, under the same condition described for cucumber, only Mn and Zn were increased in roots, while inoculation with the bacterium alone improved the allocation of Zn, Mn, and Cu to shoots. The biochemical mechanisms underlying the nutritional processes in plants and nutrient availability can be altered by PGPB as a strategy to improve plant nutrient uptake, which, in turn, is influenced by microbial strains and plant species (Pii et al., 2015b). Additionally, under equilibrate P availability, bacterial inoculation induced a decrease in the concentrations of all micronutrients in the subaerial part of cucumber plants, whilst no significant changes were induced in maize plants. Under a specific nutritional shortage, plants can also trigger physiological modulations to maintain the ionic equilibrium of the tissues (Marschner, 1995). Thus, changes in the concentration of a mineral element might induce modulations (positive or negative) of the concentration levels of one (or more than a single) essential or non-essential nutrient. It is worth noting that, under sufficient P conditions, the accumulation of Na in the roots of both plants was reduced by inoculation with *Enterobacter* 15S. Decreased concentrations of Na have been reported in plants inoculated with PGPB and AMF as a mechanism to protect plant cells against oxidative stress by maintaining intracellular ionic homeostasis (Giri and Mukerji, 2004; Panwar et al., 2016).

### A Differential Root Exudation Pattern Was Induced by P Deficiency and Bacterial Biostimulant

In specific nutritional conditions, plants can change their qualitative and quantitative composition of root exudates to increase the availability of nutrients (Badri and Vivanco, 2009) and/or to influence the microbial populations colonizing the roots (Cesco et al., 2012). Phenolic acids and flavonoids have been reported as the major secondary metabolites exudated by plant roots and are significant in diverse biological processes (Mandal et al., 2010; Cesco et al., 2012). Root exudation and regulation of these compounds seems to be an important strategy for plants to overcome P deficiency (Malus et al., 2006). Regardless of P supply, the total content of phenolic acids exuded by roots was plant species-dependent, whereas the release of flavonoids was similar in both plants hereby evaluated. In general terms, the amount and diversity of secondary metabolites released by roots are highly species-specific (Zwetsloot et al., 2018). P deficiency induced an increase in the content of phenolic acids exuded by the roots of both plants, which is in accordance with other works. For instance, under P deficiency, the amount of exuded phenolics from bean roots was five times higher (Juszczuk et al., 2004) and *Stylosanthes* roots had more than 10-fold in exudates (Luo et al., 2020), as compared to their respective controls. However, both the accumulation and exudation of phenolic compounds can also be modified in response to microbial inoculation (Vacheron et al., 2013). The inoculation of P-sufficient maize plants with *Enterobacter* 15S (treatment P+: 15SIP) induced a higher production of phenolic compounds, while the same treatment under P deficiency elicited a lower release. No effects were induced by the inoculation in cucumber plants. These results suggest that plants interacted with the bacterial biostimulant in different ways. The exudation of phenolic compounds is induced by PGPG inoculation also in plants subjected to other nutrients deficiencies. For instance, under N scarcity, peanut plants inoculated with *Stenotrophomonas maltophilia* modified their metabolism in response to the inoculum by producing a higher amount of total phenolic compounds, thus resulting in increased antioxidants and free radical scavenging activities (Alexander et al., 2019).

In the present study, we observed that the root exudation of flavonoids was lower in both plant species when compared with the total release of phenolics. P deficiency induced an increase in the flavonoids released by maize plants, while a decreased exudation of these compounds was observed in cucumber plants. Flavonoids are known for assisting plants in tolerating and overcoming biotic and abiotic stresses generated by the external environment (Shah and Smith, 2020). It has been suggested that flavonoids released from the roots of P-deficient plants can facilitate the mobilization of P-insoluble complexes (e.g., Fe-P) and also reduce the microbial degradation of organic acids (Tomasi et al., 2008). Furthermore, inoculation with PGPB can alter not only the content but also the profile of flavonoids in root exudates (Garcia-Seco et al., 2015). In this study, regardless of P supply, the inoculation with the bacterial biostimulant significantly reduced flavonoid content in the root exudates of cucumber plants, while their concentration in maize exudates was not affected, except for the treatment 15SIP (P–); in this case, a reduction of flavonoids was induced as also observed for phenolic compounds. A differential accumulation of flavonoids was described by Zuluaga et al. (2021) in root exudates of tomato plants inoculated with the same *Enterobacter* 15S strain used in the present study. In some cases, the down-accumulation of flavonoids in PGPB-inoculated plants may indicate its consumption as a C source or a blocking in their biosynthesis (Dardanelli et al., 2010; Wu et al., 2018). In this respect, it is interesting to note that the reacquisition of a fair range of exudates by roots of intact plants as recently demonstrated by Tiziani et al. (2020) makes this assessment rather complicated.

### Differential Regulation of Phosphate Transporters Was Elicited by P Starvation and the Inoculation With *Enterobacter* 15S

Members of the *PHT1* family are the most intensively studied Pi transporters genes in plants. They are known for exhibiting strong expression in the roots of both monocots and dicots (Nussaume et al., 2011). Most of these genes are strongly induced under P-limiting conditions and have been reported to be involved in the root uptake of Pi from soil solutions and then its translocation within the plant (Wang et al., 2018). Consistently, all the Pi transporters genes herein evaluated were greatly expressed in the roots of both P-starved plant species compared to P-sufficient ones. Since bacterial inoculation in P-deficient cucumber plants supplemented with Ca_3_(PO_4_)_2_ increased the P accumulation in roots and shoots, a reduction in the expression of the *CsPTs* genes compared to uninoculated controls can reasonably be expected. However, in P deficiency, all Pi transporter genes were over-expressed in cucumber plants inoculated with *Enterobacter* 15S. Moreover, *CsPT1.3* and *CsPT1.4* were also upregulated in the inoculation treatment combined with the insoluble phosphate. Indeed, the downregulation of Pi transporters by microbial inoculants has not been observed for all *PHT1* Pi transporters (Duan et al., 2015). Accordingly, the results might suggest that *Enterobacter* 15S in association with cucumber plants supplemented with Ca_3_(PO_4_)_2_ would have induced a greater P-uptake capacity. Similar behavior was also reported by Cataldi et al. (2020) in the expression levels of *TaPHT6* during the interaction of wheat plants with *Bacillus* strain 12A under P starvation. Concerning the inoculation with the bacteria alone, the results might suggest that *Enterobacter* 15S could have triggered the different gene regulation, including the modulation of molecular entities other than *PHT1* members. Indeed, although few works have studied the effects of PGPB on the regulation of Pi transporters, it is well-known that Pi acquisition and homeostasis can also be controlled by members of the *PHT2, PHT3, PHT4*, and *PHT5* families (Wang et al., 2017). Moreover, in plant-microbe interactions, in particular, in the case of AMF, AM-specific Pi transporters and AM-inducible Pi transporters identified in monocot and dicot species are essential to Pi transport (Zhang et al., 2019). Additionally, it cannot be excluded that the increased P accumulation observed in cucumber roots could also be a direct effect of the bacterium on plant metabolism, as previously reported by Saia et al. (2015) in wheat plants inoculated with the PGPB *Bacillus* species. Interestingly, in cucumber plants grown under P sufficient conditions, the expression of Pi transporters was not altered by the inoculant treatments. These findings might suggest that *Enterobacter* 15S has a better performance in dicots growing under a nutritional shortage.

On the other hand, in P-deficient maize plants, the expression of *ZmPT1* and *ZmPT5* was inhibited by inoculation with the bacterial biostimulant compared to the uninoculated controls. A similar result has been described by Liu et al. (2018) in the transcript level of *TaPT4* in wheat roots inoculated with *Pseudomonas* strain P34-L. The authors suggested that a better P nutritional state was induced by the bacterium colonizing the wheat rhizosphere (Liu et al., 2018). However, considering all the results described in the previous sections, we cannot state that the downregulation of maize Pi transporters in the inoculated P-deficient plants is due to a change in P nutrition due to PGPB interaction. However, the repression of maize Pi transporters might be triggered by the plant in response to the association with the bacterium, which could likely produce a direct effect on plant metabolism. Indeed, the fact that in inoculated maize plants under P-sufficient conditions, the *ZmPT5* gene was also downregulated might support this hypothesis. Nonetheless, since few studies have been focused on P transport in plant–PGPB interactions in P-deficiency, the mechanisms by which a specific PGPB alters the expression of Pi transporters remain unclear. Furthermore, some P transporters are also transcriptionally regulated by stimuli other than P deficiencies or microbial associations, suggesting a more complex regulatory mechanism (Gu et al., 2016).

### Clustered Heatmap of Plant Responses to Bacterial Biostimulant and P Supply

**Figure 6:**
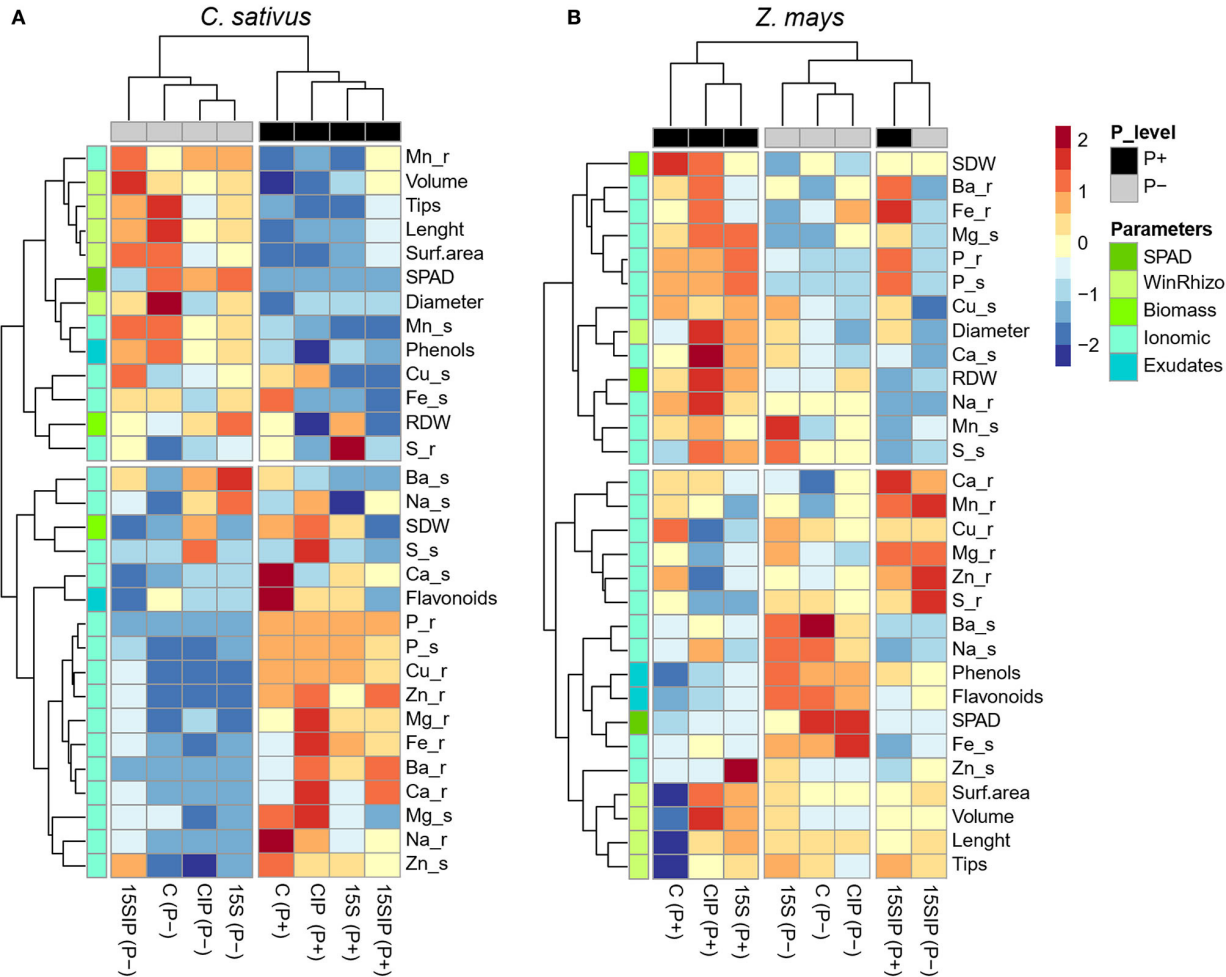
Heatmap analysis summarizing the responses of the cucumber **(A)** and maize **(B)** plants to a factorial experiment with two P-fertilization levels (P+ and P) and four biostimulant treatments (15S; 15SIP; C, uninoculated control; CIP). The heatmap was generated using the R software (https://www.r-project.org, using the “pheatmap,” “ggplot2,” and “RColorBrewer” packages). Hierarchical clustering was performed with Complete linkage and Manhattan distance was used as the similarity measure. Dark red color indicates higher relative values, while a dark-blue color reflects lower relative values. Yellow color denotes values close or equal to zero, i.e., close or equal to the mean for each parameter.

To present a visual comparison regarding the effect of inoculation with the bacterial biostimulant and levels of P supply on the growth and nutrition of cucumber and maize plants, a heatmap analysis condensing all the measured morpho-physiological, ionomic, and root exudation data was performed for each plant species (Figure 6). Both cucumber and maize plants showed different responses to the evaluated parameters. In cucumber plants, the main clustering factor responsible for different effects was P nutrition, albeit a clear effect of the bacterial biostimulant within the P levels was also observed (Figure 6A). It is expected that, under P deficiency, plants undergo various morphological, physiological, and biochemical adaptations. Intense and significant effects have been observed in cucumber plants at the levels of root system architecture, root exudation, photosynthetic rates, and nutrients under P-deficient conditions when compared to P-sufficient plants (Ciereszko et al., 2002; Zhang et al., 2012; Naureen et al., 2018). Nonetheless, the inoculation of cucumber plants with PGPB in different P states has also shown a positive contribution to the improvement of plant growth and nutrition and the abilities of the plants to deal with P deficiency (Han et al., 2006; García-López et al., 2016). The results also revealed that *Enterobacter* 15S in combination with the insoluble phosphate (15SIP) showed positive effects in alleviating the stress produced by P deprivation in cucumber plants. Since neither non-inoculated plants (CIP) nor the bacterium alone (15S) under the same conditions did not show significant effects on P nutrition, it is possible to suggest that *Enterobacter* 15S might have solubilized the Ca_3_(PO_4_)_2_ available in the dialysis tube, thus allowing cucumber plants to cope with the P limitation. Consistently, previous studies have already described the ability of *Enterobacter* strains to solubilize insoluble phosphates and promote the growth of several plant species (Ramesh et al., 2014; Bendaha and Belaouni, 2020; Zuluaga et al., 2020).

In maize plants, both P fertilization levels and biostimulant treatments affected the parameters assessed. In combination with the insoluble phosphate, *Enterobacter* 15S induced similar effects on the plant growth and nutritional state of maize plants in both P levels. However, stronger effects were induced by the bacterium alone under P-limited conditions (Figure 6B). These results suggest that, in the interaction with P-deficient maize plants, the bacterial strain *Enterobacter* 15S might have triggered different mechanisms than those activated in cucumber plants to help plants coping with P deficiency. Although all plant species can establish a relationship with some PGPB, inoculation with a specific bacterium has shown differential responses in the plant stimulation between monocots and dicots (Hall et al., 1996). However, more comparative studies between plant types and bacterial strains are still lacking. Furthermore, most existing studies are focused on PGPB and its role in improving N nutrition in plants, whereas only few pieces of literature are concerned with the effects of PGPB inoculation on plants grown in different P-supply conditions. For this reason, it has been suggested that the P-solubilization trait could be acknowledged as the forgotten child of PGP bacteria (Granada et al., 2018).

## Conclusion

The results of the present study underline those plant growth responses to inoculation with the bacterial biostimulant with *Enterobacter* 15S under specific P supplies vary between maize (a representative for monocots) and cucumber (a dicot) plants. The bacterial strain 15S induced cucumber plants to cope with P shortage by solubilizing the external source of insoluble P, which led to the modulation of root architecture, mineral nutrient uptake, root exudation of phenolics, and flavonoids, and the upregulation of P starvation-inducible Pi transporter genes. On the other hand, the ability of *Enterobacter* 15S to ameliorate the P deficiency of maize plants was less remarkable than in cucumbers, albeit the strain showed better performance under P sufficiency. These results suggest that the bacterial biostimulant strain herein evaluated has an enhanced ability to alleviate P shortage in dicots. Nonetheless, its efficiency under normal P conditions can also be extended to cultivated monocots. Still, further studies are needed to evaluate the efficacy of *Enterobacter* 15S in alleviating P nutritional shortage under different soil conditions. The need for a rapid transition of agriculture toward sustainability makes the comprehension of these phenomena even more urgent, for their use as soon as possible in the field in favor of a better use of endogenous soil resources of nutrients by crops.

## Data Availability Statement

The original contributions presented in the study are included in the article/Supplementary Material, further inquiries can be directed to the corresponding author/s.

## Author Contributions

MA, AM, YP, TM, and SC conceived the work and designed the experiment. MA, FV, and RT carried out the experiments and generated the data. MA and YP analyzed the data. MA wrote the first draft of the manuscript, which was intensively edited by all authors. YP, SC, TM, and AM reviewed the manuscript and carried out the English edition. All authors contributed to the article and approved the submitted version.

## Conflict of Interest

The authors declare that the research was conducted in the absence of any commercial or financial relationships that could be construed as a potential conflict of interest.

## Publisher's Note

All claims expressed in this article are solely those of the authors and do not necessarily represent those of their affiliated organizations, or those of the publisher, the editors and the reviewers. Any product that may be evaluated in this article, or claim that may be made by its manufacturer, is not guaranteed or endorsed by the publisher.
