# Supplementary material for: Can Inoculation With the Bacterial Biostimulant Enterobacter sp. Strain 15S Be an Approach for the Smarter P Fertilization of Maize and Cucumber Plants?
Source: Front Plant Sci. 2021 Aug 24;12:719873. doi: 10.3389/fpls.2021.719873 (PMC8421861; doi:10.3389/fpls.2021.719873)
Supplement: Supplementary file 1 [file Data_Sheet_1.PDF]

Supplementary data

Supplementary Table S1. Sequence of forward and reverse primers used in Real-time RT-PCR experiments.

Plant	Gene	Forward primer 5-3	Reverse primer 5-3	Reference
Cucumber	CsPT1.3	CGGAGGTAGAAGCCGTTGG	CATCAATCTCATAAAATATCACAA	Feil et al. 2020
	CsPT1.4	TCACTTTGTTAGTTCCTGAATC	TCAAACAAGCACAGTTCTTGA	Feil et al. 2020
	CsPT1.9	ATTCTATACACAACTACCGATG	GTGAGAAAAAAGGAAATTAAATGA	Feil et al. 2020
	Cucsa383630.1	AAAGTCATTGGAGGAGCTGAC	ACTTATACAATACAAAAATCCCC	Feil et al. 2020
	CsHA1	GAAAATGAGATAAAAGCTAAGGC	GCAAAGAACAGATGAGGAGAA	Pii et al. 2016b
	CsHA2	CGACATCGACACCATTCAGC	CTTGGCACAGCAAAGTGAAA	Pii et al. 2016b
	CsEF1a	ATTTGCTGTCCGTGATATGCG	CTTCTTCACAGCGGACTTGG	Pii et al. 2016b
Maize	ZmPT1	CACGGGTCCTCCATCCTG	CACCTGTACGAACTACTTCTG	This work
	ZmPT5	CGACCATATAAATTTGGTGCC	CACATATCACGAAGTAGTGCC	This work
	ZmHA2	AACACCTTTGCTGCCCGAC	GAAACTCCCTAGAAAGACGG	Pii et al. 2016a
	ZmHA4	TGCCACCCTTGTTGTTCTTG	TGTCTCCAATCACATCACCG	Pii et al. 2016a
	ZmEF1a	TATCTGTCTGGTGCTGTGCT	TCATAGATTACTTGTTCACGC	Pii et al. 2016a

References

Feil SB, Pii Y, Valentinuzzi F, et al (2020) Copper toxicity affects phosphorus uptake mechanisms at molecular and physiological levels in *Cucumis sativus* plants. Plant Physiol Biochem 157:138–147. https://doi.org/10.1016/j.plaphy.2020.10.023

Pii Y, Alessandrini M, Dall’Osto L, et al (2016a) Time-resolved investigation of molecular components involved in the induction of NO3 high affinity transport system in maize roots. Front Plant Sci 7:1–13. https://doi.org/10.3389/fpls.2016.01657

Pii Y, Marastoni L, Springeth C, et al (2016b) Modulation of Fe acquisition process by *Azospirillum brasilense* in cucumber plants. Environ Exp Bot 130:216–225. https://doi.org/10.1016/j.envexpbot.2016.06.011

Supplementary Table S2. ANOVA mean square values for morpho-physiological traits of cucumber (*C. sativus*) and maize (*Z. mays*) grown hydroponically for 21 days in pots filled with nutrient solution in response to two different P fertilization levels and four different inoculant treatments with the PGPB *Enterobacter* 15S.

Parameters		***Cucumber sativus***				***Zea mays***			
		Mean square[a]				Mean square[a]			
		P levels (P)[b]	Treatments (I)[c]	P × I	CV (%)	P levels (P)[b]	Treatments (I)[c]	P × I	CV (%)
**SPAD index**	SPAD	618.14***	69.54***	53.98***	2.99	103.34***	17.36***	27.01***	4.10
**WinRhizo**	Length	95,375.74***	5,382.35**	7222.54***	7.65	32,656.50***	32,909.48***	29,207.38***	6.91
	Surf area	1,095.93***	102.50***	70.77**	10.34	28.06*	467.07***	346.59***	5.02
	Diameter	0.0022**	0.0001NS	0.0005*	3.24	0.0013*	0.0002NS	0.0004NS	4.36
	Volume	0.051***	0.009***	0.001NS	13.98	0.005NS	0.029***	0.024***	8.27
	Tips	115,648.17***	13,424.44**	4,961.06*	12.77	28,773.8**	47,397.04***	37,458.04***	8.44
**Biomass**	RDW	32.43**	20.75***	11.81**	8.84	78.84*	123.92***	41.68*	14.77
	SDW	604.01**	649.25***	153.61NS	9.81	9,345.71***	1,859.47***	866.06***	7.59
	R:S ratio	0.022***	0.008***	0.0004NS	9.86	0.027***	0.029***	0.002NS	11.62

[a]Asterisks denote levels of statistical significance: * $p < 0.05$, ** $p < 0.01$, *** $p < 0.001$, NS not significant.
[b]P levels: P+ (nutrient solution supplemented with 0.1 mM KH_2PO_4); P- (nutrient solution non-supplemented with KH_2PO_4).
[c]Inoculant treatments: 15S, inoculated treatment with the PGPB *Enterobacter* 15S; 15SIP, inoculated treatment with the PGPB *Enterobacter* 15S plus the insoluble phosphate $Ca_3(PO_4)_2$; C, uninoculated control; CIP, uninoculated control with the insoluble phosphate $Ca_3(PO_4)_2$.

Supplementary Table S3. Mean values for ionomic analysis in cucumber and maize grown hydroponically under different P fertilization levels and different inoculant treatments with *Enterobacter* sp. 15S. P level effects were compared using t-tests. Differences between means were determined by Tukey's HSD test. Significant differences ($p < 0.05$) according to Tukey's test are indicated by different upper-case letters when comparing contrasts in columns and different lower-case letters when comparing contrasts in rows, and not significant differences are indicated by omitting notation letters.

**Element _ part plant**[a]	**P level**[b]	***Cucumber sativus***					***Zea mays***				
		**Inoculant treatments**[c]					**Inoculant treatments**[c]				
		C	CIP	15S	15SIP	**P level effect**	C	CIP	15S	15SIP	**P level effect**
P_r	P+	8.81 A	9.52 A	9.57 A	9.54 A	**9.36 A**	6.53 Ab	6.37 Ab	7.60 Aa	8.00 Aa	**7.13 A**
	P-	1.57 Bb	1.89 Bab	1.70 Bab	2.26 Ba	**1.86 B**	2.13 Bb	2.17 Bb	3.27 Ba	2.10 Bb	**2.42 B**
**Inoculation effect**		**5.19 b**	**5.71 ab**	**5.64 ab**	**5.90 a**		**4.33 b**	**4.27 b**	**5.43 a**	**5.05 a**	
P_s	P+	13.89 Aa	13.30 Aab	12.70 Abc	12.08 Ac	**12.99 A**	11.83 Ab	11.80 Ab	13.40 Aa	14.13 Aa	**12.79 A**
	P-	2.57 Bb	2.39 Bb	3.14 Bb	5.46 Ba	**3.39 B**	3.13 B	4.20 B	3.43 B	3.23 B	**3.50 B**
**Inoculation effect**		**8.23 ab**	**7.85 b**	**7.92 b**	**8.77 a**		**7.48 b**	**8.00 ab**	**8.42 a**	**8.68 a**	
Ca_r	P+	8.37 Ab	11.53 Aa	8.60 Ab	10.60 Aa	**9.78 A**	8.50 Ab	8.63 b	7.60 c	10.00 Aa	**8.68**
	P-	7.07 Bb	6.97 Bb	6.97 Bb	8.37 Ba	**7.34 B**	6.60 Bc	8.23 ab	7.73 bc	9.30 Ba	**7.97**
**Inoculation effect**		**7.72 b**	**9.25 a**	**7.78 b**	**9.48 a**		**7.55 c**	**8.43 b**	**7.67 c**	**9.65 a**	
Ca_s	P+	45.83 Aa	35.70 c	39.87 Ab	38.10 Abc	**39.88 A**	5.33 c	7.10 Aa	6.13 Ab	5.20 Ac	**5.94 A**
	P-	33.90 Bab	35.30 a	35.33 Ba	32.13 Bb	**34.17 B**	5.23 b	4.97 Bb	5.87 Ba	4.67 Bb	**5.18 B**
**Inoculation effect**		**39.87 a**	**35.50 c**	**37.60 b**	**35.12 c**		**5.28 b**	**6.03 a**	**6.00 a**	**4.93 c**	
Mg_r	P+	1.67 Ab	2.13 Aa	1.67 Ab	1.77 Ab	**1.81 A**	3.57 b	2.57 c	3.33 Bbc	4.50 a	**3.49**
	P-	1.10 Bc	1.33 Bab	1.13 Bbc	1.50 Ba	**1.27 B**	3.37 c	2.80 d	4.03 Ab	4.60 a	**3.70**
**Inoculation effect**		**1.38 b**	**1.73 a**	**1.40 b**	**1.63 a**		**3.47 b**	**2.68 c**	**3.68 b**	**4.55 a**	
Mg_s	P+	5.40 Aa	5.63 Aa	4.80 b	4.70 b	**5.13 A**	2.43 A	2.80 A	2.77 A	2.40 A	**2.60 A**
	P-	4.90 B	4.53 B	4.60	4.90	**4.73 B**	1.90 B	2.23 B	1.90 B	2.07 B	**2.03 B**
**Inoculation effect**		**5.15 a**	**5.08 ab**	**4.70 b**	**4.80 ab**		**2.17 b**	**2.52 a**	**2.33 ab**	**2.23 b**	
S_r	P+	11.17 Ab	6.33 c	20.43 Aa	7.00 Bc	**11.23**	7.53 a	5.60 Bb	5.43 Bb	8.03 Ba	**6.65 B**
	P-	4.63 Bb	7.70 ab	9.27 Ba	11.33 Aa	**8.23**	7.90 b	7.17 Ab	7.90 Ab	9.67 Aa	**8.16 A**
**Inoculation effect**		**7.9 bc**	**7.02 c**	**14.85 a**	**9.17 b**		**7.72 b**	**6.38 c**	**6.67 c**	**8.85 a**	
S_s	P+	5.00 c	25.13 Aa	6.80 Ab	4.13 Bc	**10.27**	2.17 ab	2.37 Aa	2.30 ab	2.13 b	**2.24**
	P-	5.70 bc	22.10 Ba	6.47 Bb	4.90 Ac	**9.79**	2.23	2.23 B	2.37	2.20	**2.26**
**Inoculation effect**		**5.35 c**	**23.62 a**	**6.63 b**	**4.52 c**		**2.20 bc**	**2.30 ab**	**2.33 a**	**2.17 c**	
Fe_r	P+	2911.13 Ac	5332.70 Aa	4377.50 Ab	5091.10 Aab	**4428.11 A**	898.43 Ab	1101.50 a	770.17 Ab	1210.10 Aa	**995.05 A**
	P-	1920.63 Bbc	1723.97 Bc	2046.50 Bb	3210.87 Ba	**2225.49 B**	765.20 Bb	1017.60 a	596.37 Bb	701.07 Bb	**770.06 B**
**Inoculation effect**		**2415.88 c**	**3528.33 b**	**3212.00 b**	**4150.98 a**		**831.82 b**	**1059.55 a**	**683.27 c**	**955.58 a**	
Fe_s	P+	192.83 Aa	127.50 Bb	130.83 Bb	118.80 Bb	**142.49 B**	79.20 B	89.00 B	81.20 B	66.93 B	**79.08 B**
	P-	168.50 Ba	140.30 Ab	173.40 Aa	173.10 Aa	**163.83 A**	106.03 Ab	126.07 Aa	111.57 Ab	82.17 Ac	**106.46 A**
**Inoculation effect**		**180.67 a**	**133.90 c**	**152.17 b**	**145.95 b**		**92.62 b**	**107.53 a**	**96.38 b**	**74.55 c**	

Zn_r	P+	189.73 Ab	215.50 Aa	150.67 Ac	223.10 Aa	**194.75 A**	320.53 Aa	180.20 Bc	236.30 b	315.43 Ba	**263.12**
	P-	51.10 Bb	55.07 Bb	56.57 Bb	119.20 Ba	**70.48 B**	247.97 Bb	267.40 Ab	260.40 b	361.00 Aa	**284.19**
**Inoculation effect**		**120.42 c**	**135.28 b**	**103.62 d**	**171.15 a**		**284.25 b**	**223.80 c**	**248.35 bc**	**338.22 a**	
Zn_s	P+	131.10 Aa	119.20 Aab	119.83 Aab	113.27 Bb	**120.85 A**	80.93 b	75.60 b	131.47 Aa	68.03 Bb	**89.01**
	P-	90.33 Bb	87.87 Bb	99.23 Bb	128.63 Aa	**101.52 B**	80.53 bc	78.10 c	92.37 Ba	89.87 Aab	**85.22**
**Inoculation effect**		**110.72 b**	**103.53 b**	**109.53 b**	**120.95 a**		**80.73 b**	**76.85 b**	**111.92 a**	**78.95 b**	
Mn_r	P+	22.00 Bb	31.60 Bb	23.50 Bb	76.30 Ba	**38.35 B**	325.40 Aa	286.03 Bb	236.50 Bc	348.53 a	**299.12**
	P-	73.57 Ab	94.67 Aa	93.47 Aa	103.03 Aa	**91.18 A**	234.67 Bb	297.27 Ab	292.30 Ab	372.00 a	**299.06**
**Inoculation effect**		**47.78 c**	**63.13 b**	**58.48 b**	**89.67 a**		**280.03 b**	**291.65 b**	**264.40 b**	**360.27 a**	
Mn_s	P+	85.77 Ba	78.83 Bab	62.40.Bb	65.00 Bb	**73.00 B**	82.83 Aa	89.30 a	77.80 Ba	56.33 Bb	**76.57**
	P-	152.10 Aa	117.57 Ac	130.07 Ab	160.23 Aa	**139.99 A**	63.90 Bc	78.40 b	103.70 Aa	71.63 Abc	**79.41**
**Inoculation effect**		**118.93 a**	**98.20 b**	**96.23 b**	**112.62 a**		**73.37 b**	**83.85 a**	**90.75 a**	**63.98 b**	
Cu_r	P+	53.30 A	59.20 A	57.43 A	48.10	**54.56 A**	65.23 Aa	38.17 Bd	45.00 Bc	59.03 b	**51.86**
	P-	16.57 Bb	16.83 Bb	19.23 Bb	39.13 a	**22.94 B**	58.30 B	52.70 A	60.20 A	56.10	**56.83**
**Inoculation effect**		**35.03**	**38.02**	**38.33**	**43.62**		**61.77 a**	**45.43 c**	**52.60 b**	**57.57 ab**	
Cu_s	P+	18.73 ab	19.13 a	14.67 Bbc	14.43 Bc	**16.74**	15.20 A	14.70 A	15.37	14.90 A	**15.04 A**
	P-	16.13 b	16.73 b	17.77 Aab	20.37 Aa	**17.75**	13.67 Bb	12.97 Bb	15.23 a	12.10 Bc	**13.49 B**
**Inoculation effect**		**17.43**	**17.93**	**16.22**	**17.40**		**14.43 b**	**13.83 bc**	**15.30 a**	**13.50 c**	
Ba_r	P+	5.20 Ac	10.10 Aa	7.30 Abc	9.50 Aab	**8.03 A**	3.10 Ab	3.77 Aa	2.30 c	3.90 Aa	**3.27 A**
	P-	1.73 Bb	1.67 Bb	1.77 Bb	2.70 Ba	**1.97 B**	1.53 Bb	2.43 Ba	2.40 a	1.57 Bb	**1.98 B**
**Inoculation effect**		**3.47 b**	**5.88 a**	**4.53 b**	**6.10 a**		**2.32 c**	**3.10 a**	**2.35 c**	**2.73 b**	
Ba_s	P+	2.60 Aa	2.13 Bab	1.97 Bb	2.10 Bab	**2.20 B**	0.67 B	0.70	0.67 B	0.60	**0.66 B**
	P-	2.00 Bb	2.77 Aa	3.13 Aa	2.60 Aab	**2.63 A**	1.03 Aa	0.77 b	0.93 Aa	0.63 b	**0.84 A**
**Inoculation effect**		**2.30**	**2.45**	**2.55**	**2.35**		**0.85 a**	**0.73 ab**	**0.80 a**	**0.62 b**	
Na_r	P+	6.70 Aa	4.90 Ab	3.40 Ac	4.03 Abc	**4.76 A**	4.30 Ab	5.07 Aa	3.80 b	2.60 c	**3.94 A**
	P-	2.00 Bb	1.93 Bb	2.23 Bb	3.20 Ba	**2.34 B**	3.40 Ba	3.50 Ba	3.53 a	2.53 b	**3.24 B**
**Inoculation effect**		**4.35 a**	**3.42 bc**	**2.82 c**	**3.62 b**		**3.85 a**	**4.28 a**	**3.67 b**	**2.57 c**	
Na_s	P+	2.40 Ab	2.83 a	1.87 Bc	2.50 ab	**2.40**	1.67 Bb	2.40 a	1.53 Bb	1.37 b	**1.74 B**
	P-	2.07 Bb	2.70 a	2.93 Aa	2.50 ab	**2.55**	2.53 Aa	2.13 b	2.63 Aa	1.40 c	**2.18 A**
**Inoculation effect**		**2.23b**	**2.77 Aa**	**2.40 b**	**2.50 ab**		**2.10 a**	**2.27 a**	**2.08 a**	**1.38 b**	

[a] Elements content in roots (r) or shoots (s): P, phosphorus (mg g^{-1} part plant); Ca, calcium (mg g^{-1} part plant); Mg, magnesium (mg g^{-1} part plant); S, sulfur (mg g^{-1} part plant); Fe, iron (µg g^{-1} part plant); Zn, zinc (µg g^{-1} part plant); Mn, manganese (µg g^{-1} part plant); Cu, copper (µg g^{-1} part plant), Ba, barium (µg g^{-1} part plant); Na, sodium (mg g^{-1} part plant).

[b] P levels: P+ (nutrient solution supplemented with 0.1 mM KH_2PO_4); P- (nutrient solution non-supplemented with 0.1 mM KH_2PO_4).

[c] Inoculant treatments: C, uninoculated control; CIP, uninoculated control with the insoluble phosphate $Ca_3(PO_4)_2$; 15S, inoculated treatment with the PGPB *Enterobacter* sp. 15S; 15SIP, inoculated treatment with the PGPB *Enterobacter* sp. 15S plus the insoluble phosphate $Ca_3(PO_4)_2$.
